# A Simple Remedy

**Published:** 1884-02

**Authors:** 


### HALL'S JOURNAL OF HEALTH AND MISCELLANY.

MONTHLY.

E. H. GIBBS, A. M., M. D., EDITOR.

FOUNDED IN 1854.

### A SIMPLE REMEDY.

THERE is no remedy of such general application, and none so easily attainable, as water; and yet nine persons in ten will pass by it in an emergency to seek for something of far less efficiency.

There are but few cases of illness where water should not occupy the highest place as a remedial agent.

A strip of flannel or a napkin folded lengthwise, and dipped in hot water and wrung out, and then applied around the neck of a child that has croup, will usually bring relief in ten minutes.

A towel folded several times, and dipped in hot water and quickly wrung and applied over the seat of the pain in toothache or neuralgia, will generally afford prompt relief. This treatment in colic works almost like magic. I have seen cases that have resisted other treatment for hours yield to this in ten minutes. There is nothing that will so promptly cut short a congestion of the lungs, sore throat, or rheumatism, as hot water when applied promptly and thoroughly.

Pieces of cotton batting dipped in hot water, and kept applied to old sores or new, cuts, bruises or sprains, is the treatment now generally adopted in hospitals. I have seen a sprained ankle cured in an hour by showering it with hot water, poured from a height of three feet.

Tepid water acts promptly as an emetic; and hot water taken freely half an hour before bed time is the best of cathartics in cases of constipation, while it has a most soothing effect on the stomach and bowels. This treatment continued for a few months, with proper attention to the diet, will cure any curable case of dyspepsia.

Headache almost always yields to the simultaneous application of hot water to the feet and the back of the neck.

It is an excellent plan to record facts like these in a note book, which should be always at hand when wanted. In the anxiety caused by accidents or sudden illness in the family, one becomes confused and is not apt to remember quickly what should be done; hence there may be prolonged and unnecessary suffering before proper remedies are applied.

